# Epstein-Barr virus lytic gene BNRF1 promotes B-cell lymphomagenesis via IFI27 upregulation

**DOI:** 10.1371/journal.ppat.1011954

**Published:** 2024-02-01

**Authors:** Ken Sagou, Yoshitaka Sato, Yusuke Okuno, Takahiro Watanabe, Tomoki Inagaki, Yashiro Motooka, Shinya Toyokuni, Takayuki Murata, Hitoshi Kiyoi, Hiroshi Kimura

**Affiliations:** 1 Department of Virology, Nagoya University Graduate School of Medicine, Nagoya, Japan; 2 Department of Hematology and Oncology, Nagoya University Graduate School of Medicine, Nagoya, Japan; 3 Department of Virology, Nagoya City University Graduate School of Medical Sciences, Nagoya, Japan; 4 Department of Pathology and Biological Responses, Nagoya University Graduate School of Medicine, Nagoya, Japan; 5 Department of Virology, Fujita Health University School of Medicine, Toyoake, Japan; University of North Carolina at Chapel Hill, UNITED STATES

## Abstract

Epstein-Barr virus (EBV) is a ubiquitous human lymphotropic herpesvirus that is causally associated with several malignancies. In addition to latent factors, lytic replication contributes to cancer development. In this study, we examined whether the lytic gene BNRF1, which is conserved among gamma-herpesviruses, has an important role in lymphomagenesis. We found that lymphoblastoid cell lines (LCLs) established by BNRF1-knockout EBV exhibited remarkably lower pathogenicity in a mice xenograft model than LCLs produced by wild-type EBV (LCLs-WT). RNA-seq analyses revealed that BNRF1 elicited the expression of interferon-inducible protein 27 (IFI27), which promotes cell proliferation. IFI27 knockdown in LCLs-WT resulted in excessive production of reactive oxygen species, leading to cell death and significantly decreased their pathogenicity *in vivo*. We also confirmed that IFI27 was upregulated during primary infection in B-cells. Our findings revealed that BNRF1 promoted robust proliferation of the B-cells that were transformed by EBV latent infection via IFI27 upregulation both *in vitro* and *in vivo*.

## Author summary

Virus-infected cells must overcome several anti-viral host responses for stable growth. The human oncogenic herpesvirus Epstein-Barr virus (EBV) carries the genes with anti-apoptotic and proliferative functions. In this study, we demonstrated the role of the EBV protein BNRF1 in the growth resilience of B-cells infected with EBV. BNRF1 induced the expression of interferon-inducible protein 27 (IFI27), driving the proliferation of infected cells. IFI27 knockdown elicited the over production of reactive oxygen species, causing a fragile growth of both EBV-infected and EBV-transformed cells. Furthermore, disruption of the BNRF1-IFI27 axis reduced the pathogenicity of lymphoblastoid cell lines in a mouse xenograft model. These results provide insights into the neoplastic progression of EBV-infected cells and therapeutic targets against EBV-infected cells.

## Introduction

Epstein-Barr virus (EBV) is a ubiquitous human lymphotropic herpesvirus that is causally associated with several malignancies including Burkitt lymphoma, Hodgkin lymphoma, a part of diffuse large B-cell lymphoma (DLBCL), post-transplant lymphoproliferative disorders, T/NK cell lymphoma, and nasopharyngeal carcinoma [1,2]. EBV establishes latent infection in B cells, in which the virus expresses latent factors rather than producing infectious particles. These EBV factors transform primary B cells into lymphoblastoid cell lines (LCLs) *in vitro*. EBV-mediated suppression of apoptosis plays critical roles in LCL growth and survival [3]. In addition to latent factors, accumulating evidence indicates that lytic replication, the process that generates new virus progeny by viral lytic proteins, contributes to cancer development [4–6].

The EBV tegument protein BNRF1 is an abundant protein in the virion [7], and it exerts multiple effects. BNRF1 homologs are present in all gamma-herpesviruses such as KSHV ORF75 [8] but absent in alpha- and beta-herpesviruses. BNRF1 disrupts ATRX/Daxx complexes to prevent the loading of repressive H3.3 histones onto incoming EBV genomes [9]. BNRF1 knockout (KO) impairs the expression of EBNA2 during the earliest stages of EBV infection in B-cells [9,10]. BNRF1 enables efficient viral replication by targeting SMC5/6 cohesin complexes to a ubiquitin-proteasome pathway [11]. Furthermore, BNRF1 induces centrosome amplification, leading to chromosomal instability even without establishing chronic infection [12]. Although BNRF1-mediated chromosomal instability is expected to contribute to the initial development of cancer [13], the role of BNRF1 in oncogenesis *in vivo* remains unclear.

In this study, we found that LCLs established by BNRF1-KO EBV exhibited remarkably lower pathogenicity in a mice xenograft model than LCLs produced using wild-type EBV (LCLs-WT). BNRF1 elicited the expression of interferon-inducible protein 27 (IFI27), which promotes cell proliferation [14–19]. The knockdown of IFI27 in LCLs-WT significantly reduced their pathogenicity *in vivo*.

## Results

### BNRF1 enhanced the frequency of tumor formation in a mouse xenograft model

**Fig 1:**
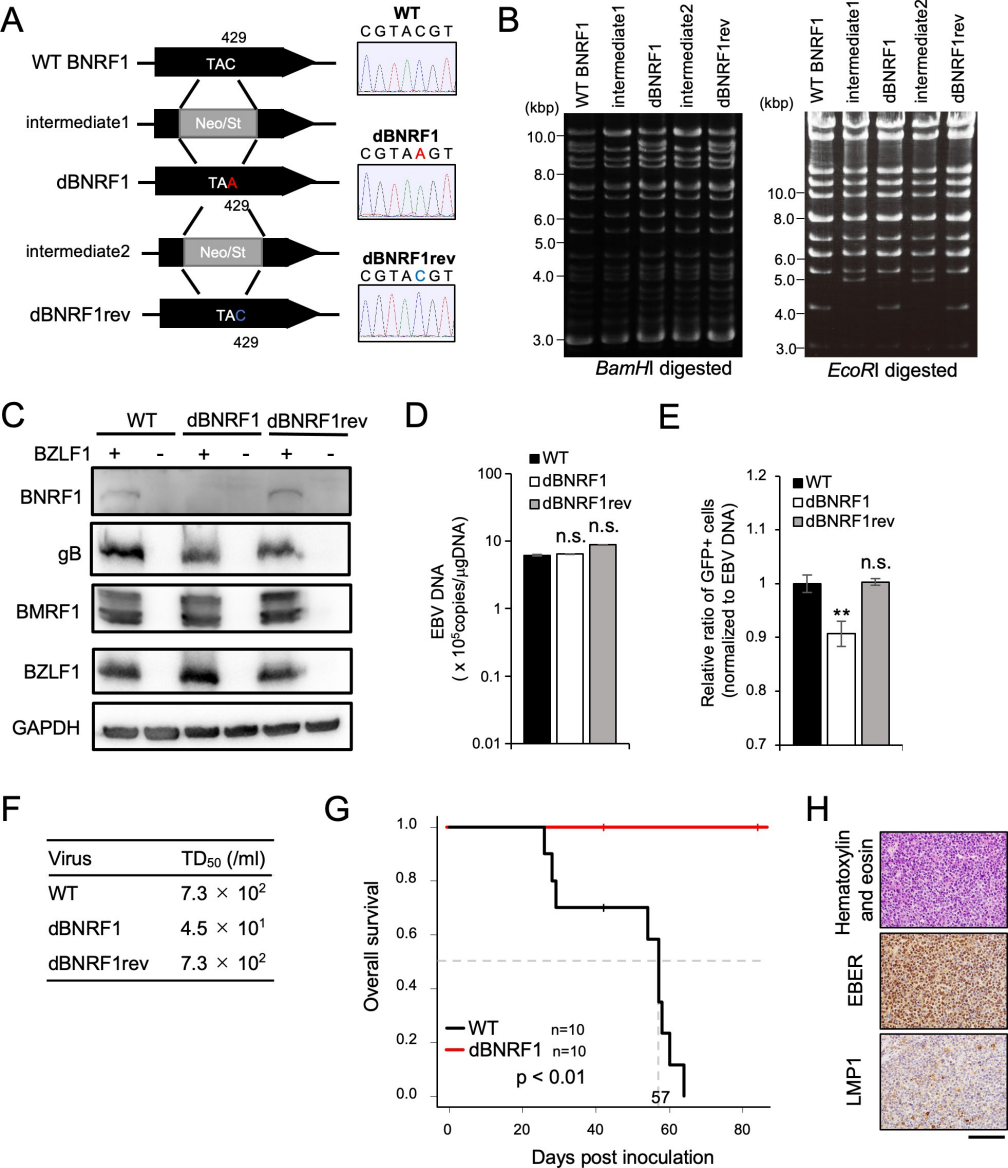
Characterization of BNRF1-KO EBV. (A) Schematic diagrams of BNRF1-KO recombinant viruses used in this study. The Neo/St cassette containing the neomycin resistance and streptomycin sensitivity genes was inserted between nucleotides 312 and 738 of the BNRF1 gene to prepare an intermediate, and the C429A (stop codon) mutation was introduced when this cassette was removed (dBNRF1). The Neo/St cassette was reinserted to the same position of dBNRF1 (intermediate 2), and then A429 was replaced with C when this cassette was removed (dBNRF1rev). Successful recombination was confirmed by Sanger sequencing as presented in the right panels. (B) Electrophoresis of EBV-BAC digested with *BamH*I (left) or *EcoR*I (right). (C) Immunoblots of lysates from HEK293T cells carrying the indicated EBV-BAC with or without pcDNA-BZLF1 transfection with the indicated antibodies. (D) Quantification of viral genomic DNA in HEK293T cells carrying the indicated recombinant EBV-BAC genomes at 72 h after pcDNA-BZLF1 transfection. The results in the bar graphs are presented as the mean ± SD. n.s., not significant. (E) Virus titers in 100 μL of supernatants were determined by counting the proportion of EGFP-positive Akata(-) cells by flow cytometry 2 days after infection. The results in the bar graphs are presented as the mean ± SD. ** p < 0.01 compared to WT. (F) PBMCs were infected with 10-fold serial dilutions of the indicated EBV. After 3 weeks, the transformation efficiency (TD_50_/mL) was calculated by examining the number of wells in which clumps of LCLs were present. (G) The pathogenicity of LCLs in vivo. Overall survival for 6-week-old mice inoculated with LCLs-WT or LCLs-dBNRF1. The 50% survival was 57 days in LCLs-WT. (H) Histochemistry of the intraperitoneal tumors stained with hematoxylin and eosin (top), and analyzed by EBER in situ hybridization (middle) and LMP1 immunohistochemistry (bottom). The images are representative of two independent experiments with similar results. Scale bar, 100 μm.

To elucidate the roles of BNRF1 in tumor formation, we first generated a BNRF1-KO mutant-BAC (dBNRF1-rEBV) and revertant EBV-BAC (dBNRF1rev-rEBV) from WT EBV-BAC (WT-rEBV), as presented in Fig 1A. These EBV-BACs were analyzed by Sanger sequencing and restriction digestion with *BamH*I or *EcoR*I, followed by agarose gel electrophoresis (Fig 1A and 1B). We performed these full bacmid sequencing by Nanopore technology and confirmed no off-target mutation among these rEBVs (S1 Fig). Subsequently, we established HEK293T/WT-rEBV, HEK293T/dBNRF1-rEBV, and HEK293T/dBNRF1rev-rEBV cells carrying each recombinant EBV, and viruses produced from these cells were named EBV-WT, EBV-dBNRF1, and EBV-dBNRF1rev, respectively.

BNRF1 KO was also confirmed by immunoblotting (Fig 1C). The expression of glycoprotein B, a late gene, was not affected by the introduction of a stop mutation in the BNRF1 gene (Fig 1C). BNRF1 KO did not affect viral DNA synthesis (Fig 1D). Consistent with previous reports [9,10], the infectivity of the BNRF1-KO virus was significantly lower than that of EBV-WT and EBV-dBNRF1rev after normalization to the EBV DNA copy number (Fig 1E). The transformation efficiency of EBV-dBNRF1 was approximately 80-fold lower than those of EBV-WT and EBV-dBNRF1rev (Fig 1F). These findings coincided with the reported phenotype of the EBV mutant lacking the BNRF1 gene [10].

BNRF1 protein induces chromosomal instability via centrosome amplification without establishing a chronic infection [12], suggesting its contribution to tumor development. However, the role of BNRF1 in pathogenesis *in vivo* remains obscure. To examine this, we established LCLs via recombinant EBV-WT or EBV-dBNRF1 infection of peripheral blood mononuclear cells (PBMCs) isolated from a healthy donor (LCLs-WT and LCLs-dBNRF1, respectively) and then evaluated these LCLs in an *in vivo* mouse model of B-cell lymphoma [20]. When injected intraperitoneally into 6-week-old NOD/Shi-scid-IL2Rγ^null^ immunodeficient mice (NOG) mice, LCLs-dBNRF1 exhibited remarkably lower pathogenicity than LCLs-WT. LCLs-dBNRF1 did not form lymphomas, and all mice survived until day 70 after inoculation (Fig 1G). The tumors that developed in LCLs-WT-injected mice expressed LMP1 and EBER (Fig 1H). Interestingly, the viral load in the blood of mice with LCLs-dBNRF1 was detected at 42 days post-inoculation but at low levels, and subsequently tapered at 77 days post-inoculation (S2 Fig). These findings imply that disruption of the BNRF1 gene abrogated the pathogenicity of LCLs *in vivo* due to the fragile growth of LCLs-dBNRF1. It should be noted that LCLs-dBNRF1 formed tumors in 5-week-old NOG mice (S3 Fig).

### Loss of BNRF1 reduced LCL survival

**Fig 2:**
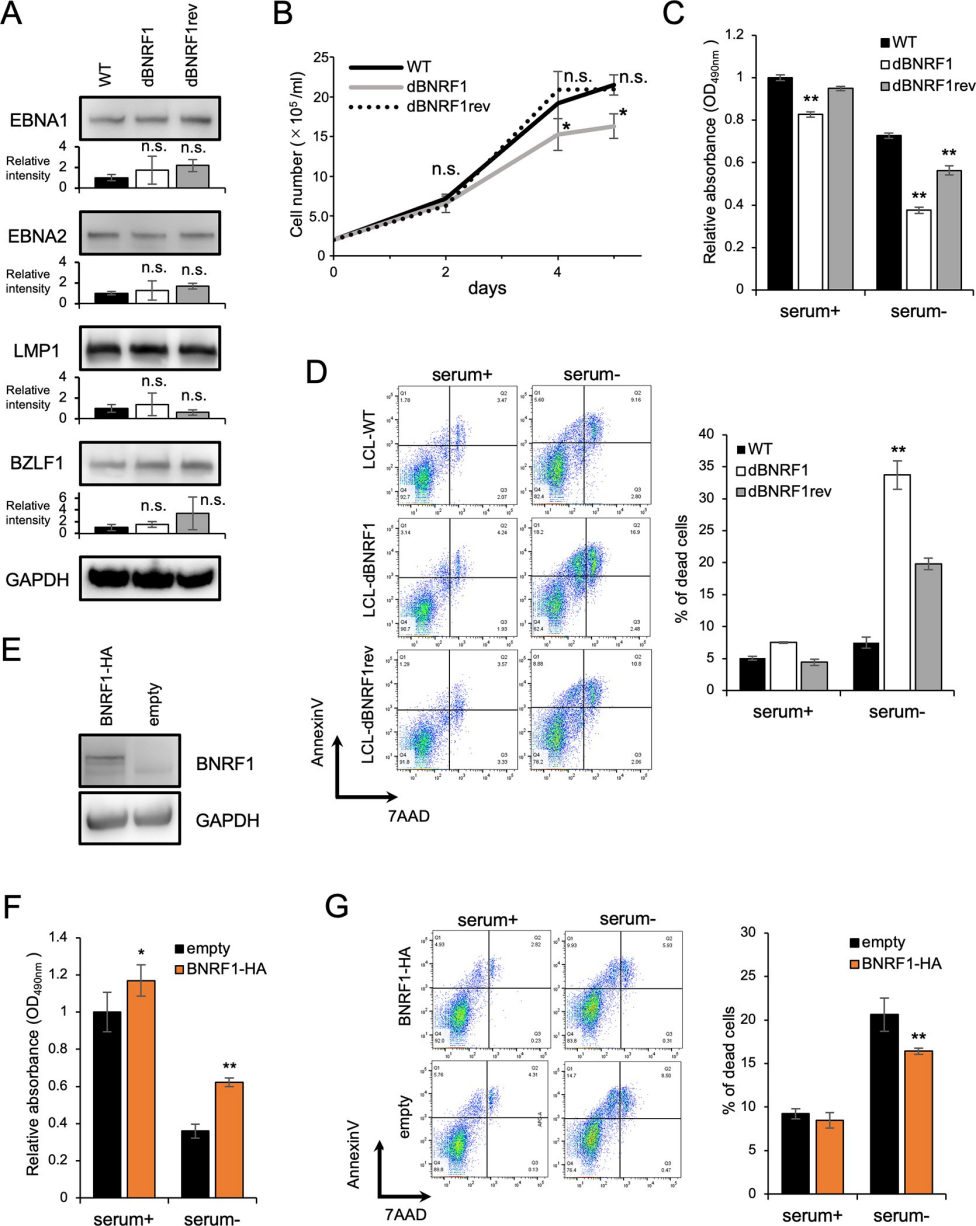
LCL-dBNRF1 exhibited slower growth than LCLs-WT. (A) Immunoblots of lysates from LCLs-WT, LCLs-dBNRF1, and LCLs-dBNRF1rev with the indicated antibodies. The results in the bar graphs are presented as the relative mean intensity ± SD. n.s., not significant. (B) The growth curves of the indicated LCLs over 5 days after seeding at 2 × 10^5^ cells. The results are presented as the mean ± SD of three independent experiments. * p < 0.05 compared to LCLs-WT. (C) Viability of LCLs cultured with or without serum for 24 h as assessed by the MTS assay. The absorbance at 490 nm is normalized to LCLs-WT cultured in the presence of serum. The results are presented as the mean ± SD. ** p < 0.01 compared to any LCLs with the same condition. (D) Annexin V/7-AAD assay of LCLs with or without serum. LCLs were maintained with or without serum-depleted medium for 24 h and then harvested. Dead cells were defined as those positive for annexin V or both annexin V and 7-AAD. The results in the bar graphs are presented as the mean ± SD. ** p < 0.01 compared to any LCLs without serum. (E) Immunoblots confirming the trans-complementation of BNRF1 in LCLs-dBNRF1. (F) Viability of LCLs-dBNRF1 complemented with BNRF1 with or without serum for 24 h as assessed by the MTS assay. The results are presented as the mean ± SD. * p < 0.05, ** p < 0.01. (G) Annexin V/7-AAD assay of LCLs-dBNRF1 complemented with BNRF1 with or without serum. Dead cells were defined as those positive for annexin V or both annexin V and 7-AAD. The results are presented as the mean ± SD. ** p < 0.01.

Next, we characterized each LCL *in vitro*. EBNA1, EBNA2, LMP1, and BZLF1 expression did not significantly differ among LCLs-WT, LCLs-dBNRF1, and LCLs produced by EBV-dBNRF1rev (LCLs-dBNRF1rev; Fig 2A). Of note, the numbers BZLF1^+^ and BZLF1^+^/glycoprotein B^+^ cells showing spontaneous lytic reactivation [21] were comparable among these LCLs (S1 Table).

However, the growth rate of LCLs-dBNRF1 was significantly lower than those of LCLs-WT and LCLs-dBNRF1rev (Fig 2B). We further assessed the growth properties of LCLs-dBNRF1 with or without serum deprivation using the 3-(4,5-dimethylthiazol-2-yl)-5-(3-carboxymethoxyphenyl)-2-(4-sulfophenyl)-2H-tetrazolium, inner salt (MTS) assay and annexin V/7-aminoactinomycin D (7-AAD) (Fig 2C and 2D, respectively). As shown in Fig 2C, LCLs-dBNRF1 exhibited growth delay. Serum deprivation enhanced this growth phenotype (Fig 2C). The annexin V/ 7-AAD assay revealed that BNRF1-KO increased cell death (Fig 2D).

To examine whether BNRF1 is responsible for this phenotype of LCLs-dBNRF1, we performed trans-complementation analyses. Exogenous HA-tagged BNRF1 was expressed in LCLs-dBNRF1 via lentivirus-mediated transduction (Fig 2E). The exogenous expression of BNRF1-HA enhanced cell proliferation (Fig 2F) and decreased cell death under serum deprivation (Fig 2G), indicating the pivotal role of BNRF1 in LCL growth.

### Expression of the BNRF1 gene in LCLs

**Fig 3:**
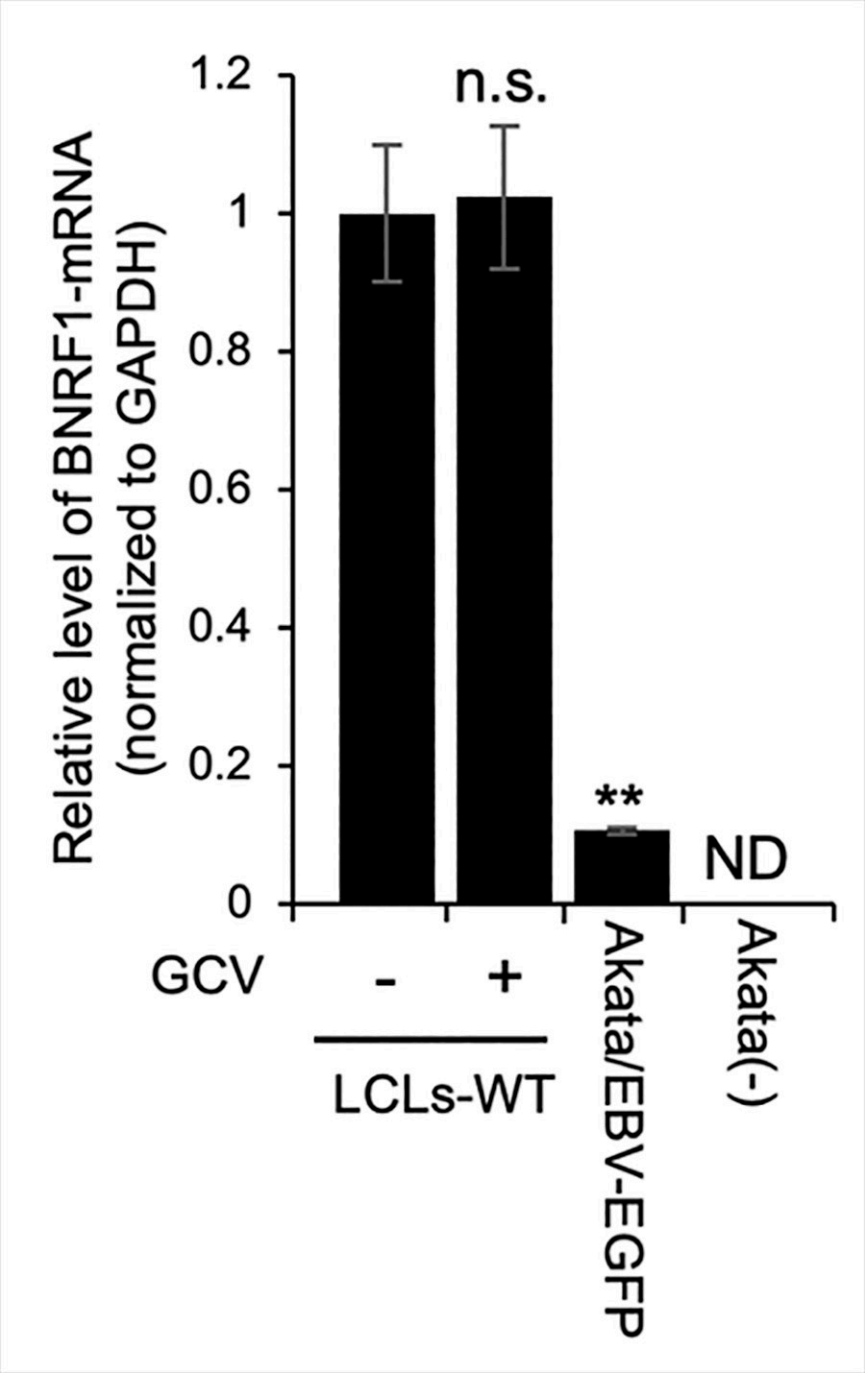
LCLs express BNRF1-mRNA in the latent state. LCLs-WT was cultured for 48 h with or without 20 μM ganciclovir (GCV). Total RNA extracted from the indicated LCLs-WT, Akata/EBV-EGFP cells, and Akata(-) cells was examined by RT-qPCR. The results are presented as the mean ± SD. ** p < 0.01, n.s., not significant, ND, not detected.

To investigate the expression of BNRF1 in LCLs, we performed immunoblotting with the anti-BNRF1 antibody. As shown in S4 Fig, we could not detect the protein encoded by BNRF1. However, owing to the low sensitivity of the antibody, the possibility that BNRF1 is expressed in LCLs cannot be excluded. Thus, we measured the levels of RNA encoding BNRF1 using quantitative real-time reverse transcription-PCR (RT-qPCR) analysis and detected BNRF1-mRNA in LCLs (Fig 3). Lytic replication is spontaneously detected in a small fraction of LCLs. To address the expression of BNRF1 in the B-cells latently infected with EBV, LCLs were treated with ganciclovir to inhibit the lytic replication of EBV [22]. Equal levels of BNRF1-mRNA were detected with or without ganciclovir treatment (Fig 3), suggesting that BNRF1 was expressed in most LCLs rather than in small population supporting the lytic cycle.

Compared with LCLs, BNRF1 expression was lower in Akata/EBV-EGFP cells (Fig 3). The growth of Akata/EBV-EGFP was not dependent on EBV, because its parental Akata(-) cell is a cell line established from Burkitt lymphoma. These findings support the role of BNRF1 in overcoming fragile growth.

### BNRF1 induced IFI27 expression

**Fig 4:**
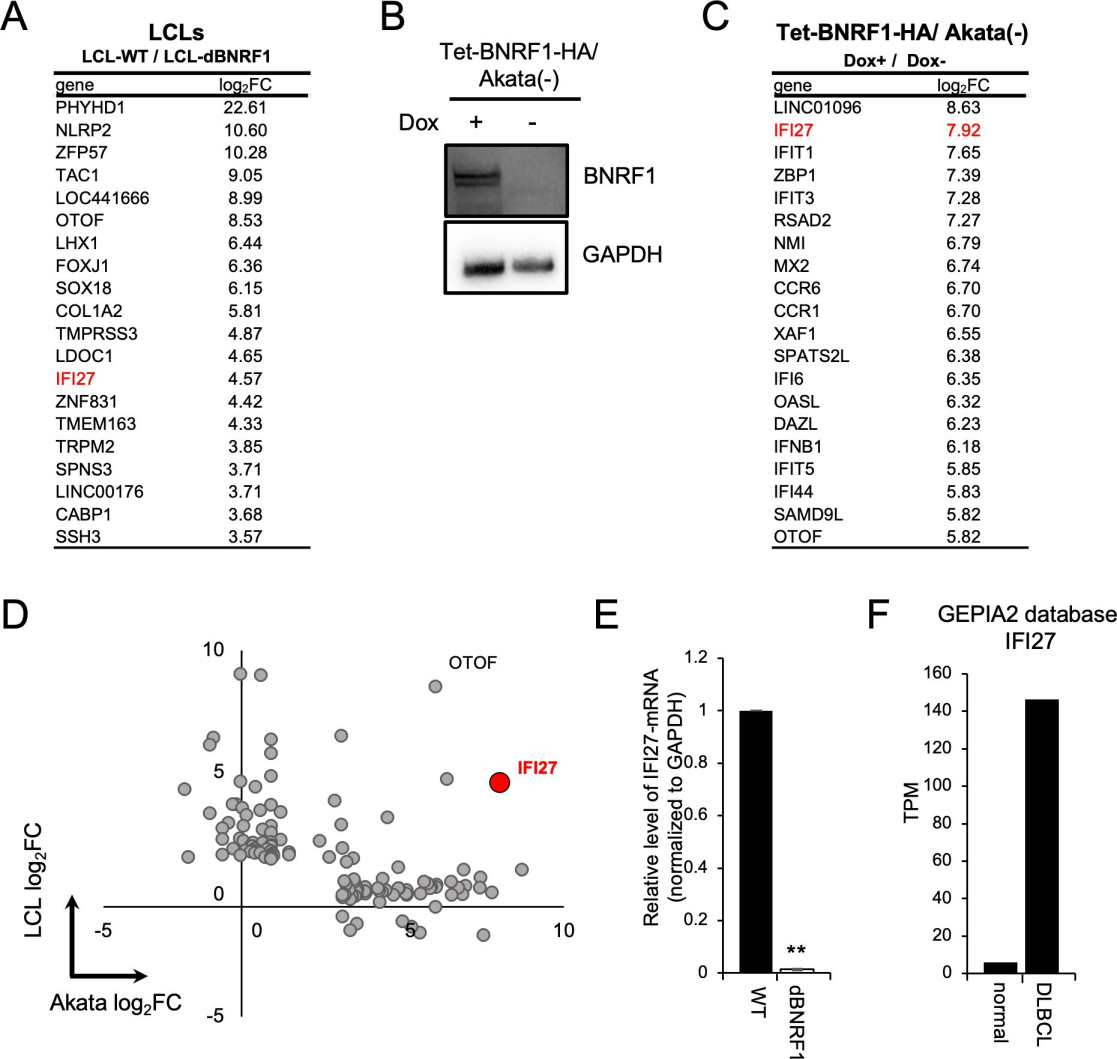
RNA-seq analyses of LCLs and Akata(-) cells inducibly expressing BNRF1. (A) Upregulated genes in LCLs-WT compared to those in LCLs-dBNRF1 as determined using the log_2_ fold-change (FC). The top 20 genes are presented. (B) Western blotting confirming BNRF1 expression in Tet-BNRF1/Akata(-) cells after the addition of Dox. (C) Upregulated genes in Tet-BNRF1/Akata(-) cells treated with Dox compared to untreated cells as determined using log_2_ FC. The top 20 genes are presented. (D) Top 120 genes upregulated in LCLs-WT and Tet-BNRF1/Akata(-) cells with Dox are shown in the scatter plot of log_2_ FC in LCLs and Tet-BNRF1/Akata(-) cells. (E) Validation of IFI27 mRNA expression in LCLs. Total RNA extracted from the indicated LCLs was examined by RT-qPCR. The results are presented as the mean ± SD. ** p < 0.01. (F) IFI27 mRNA expression in diffuse large B cell lymphoma according to RNA-seq data in the GEPIA2 database.

To elucidate the mechanisms underlying the BNRF1-mediated growth advantage of LCLs, we compared gene expression profiles between LCLs-WT and LCLs-dBNRF1. The upregulated genes are listed in Fig 4A and S2 Table. Consistent with our findings in Fig 2, GO term analysis showed that the growth was stimulated in LCLs-WT compared with LCLs-dBNRF1 (S5 Fig). Of note, obvious differences in EBV gene expression were not observed between LCLs-WT and LCLs-dBNRF1 (S3 Table).

To decrease bias and further narrow BNRF1-responsive genes, we established Akata(-) cells expressing HA-tagged BNRF1 in a tetracycline-inducible manner (Tet-BNRF1-HA/Akata(-) cells; Fig 4B) and then performed RNA-seq analysis using Tet-BNRF1-HA/Akata(-) cells with or without doxycycline (Dox) induction (Fig 4C and S4 Table). As illustrated in Fig 4D, IFI27 was universally selected as a BNRF1-responsive gene in both BNRF1-KO LCLs and BNRF1-expressing Akata(-) cells. Otofelin (OTOF) was excluded as a candidate because of its low expression in both LCLs and Akata(-) cells. We validated the elevated expression level of IFI27 in LCLs-WT compared to that in LCLs-dBNRF1 by RT-qPCR (Fig 4E).

In addition, our previous time-course analysis of RNA-seq data from PBMCs infected with wild-type EBV indicated that the mRNA expression of BNRF1 and IFI27 similarly elevated from 4 dpi (S6 Fig) [23]. It should be noted that IFI27 is upregulated in clinical samples isolated from patients with DLBCL, which is sometimes associated with EBV (GEPIA2 database [24]; Fig 4F).

**Fig 5:**
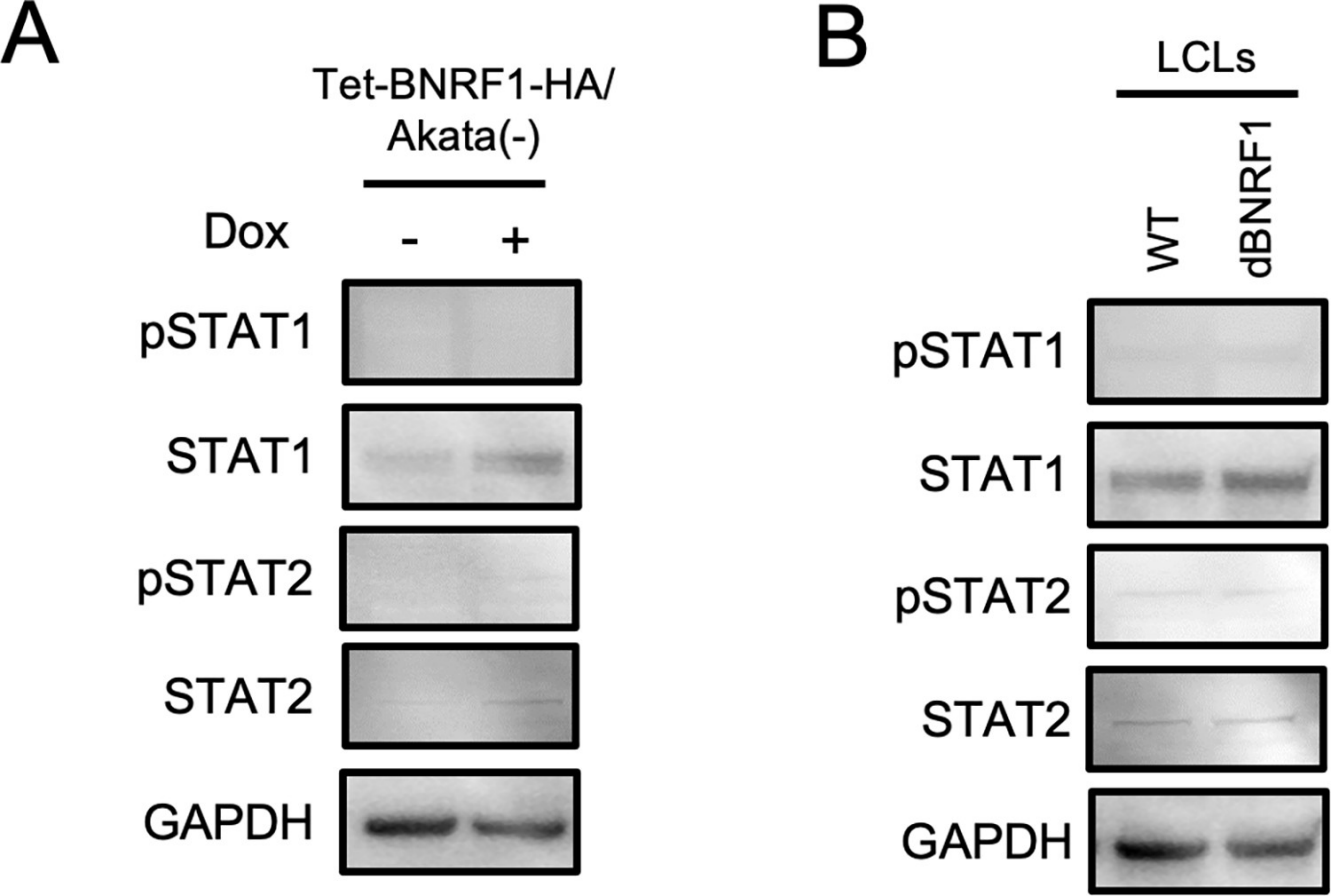
The expression of STAT1 and STAT2 in Akata(-) cells inducibly expressing BNRF1 and LCLs. (A) Immunoblots of lysates from Tet-BNRF1/Akata(-) cells cultured with or without Dox with the indicated antibodies. (B) Immunoblots of lysates from LCLs-WT and LCLs-dBNRF1 with the indicated antibodies.

A previous study revealed that the expression of IFI27 was induced by STAT1, independent of the STAT1 phosphorylation [25]. We confirmed that BNRF1 upregulated STAT1 in Akata(-) cells (Fig 5A). However, several EBV latent proteins can upregulate STAT1 [26,27] to maintain the latency in EBV-transformed cells [28]. As shown in Fig 5B, LCLs-WT and LCLs-dBNRF1 express STAT1 to the same level, suggesting that the mechanism for the downregulation of IFI27 by BNRF1-KO has. To uncover the mechanisms, further study is required.

### IFI27 enhanced the survival of LCLs-dBNRF1

**Fig 6:**
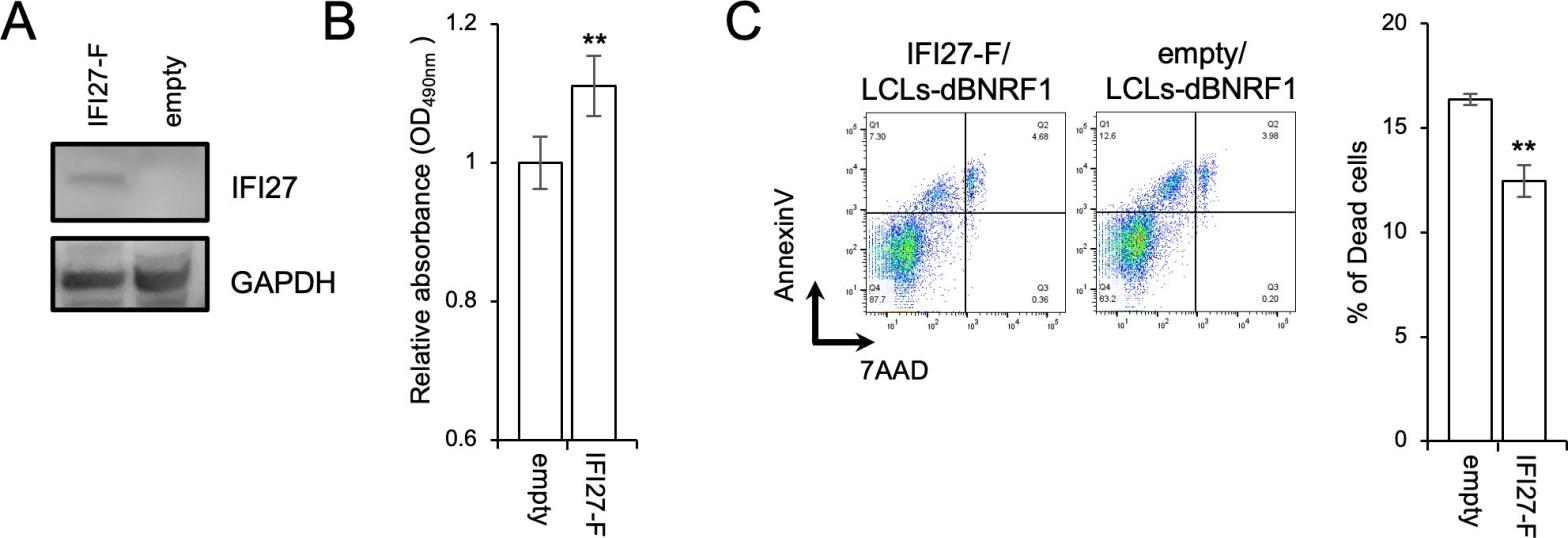
IFI27 supports LCL survival. (A) Immunoblots confirming the expression of IFI27 tagged with a FLAG epitope (IFI27-F) in LCLs-dBNRF1. (B) Viability of LCLs-dBNRF1 with or without exogenous IFI27 cultured for 48 h as assessed by the MTS assay. The results are presented as the mean ± SD. ** p < 0.01. (C) Annexin V/7-AAD assay of LCLs-dBNRF1 with or without exogenous IFI27. Dead LCLs were defined as those positive for annexin V or both annexin V and 7-AAD. The results are presented as the mean ± SD. ** p < 0.01.

To investigate the impact of IFI27 on LCLs, IFI27 was transduced into LCLs-dBNRF1 using lentiviral vectors, and IFI27-expressing cells were selected with blasticidin (Fig 6A). IFI27 overexpression in LCLs-dBNRF1 significantly stimulated cell proliferation and decreased the rate of cell death (Fig 6B and 6C).

### IFI27 knockdown reduced the pathogenicity of LCLs in a mouse xenograft model

**Fig 7:**
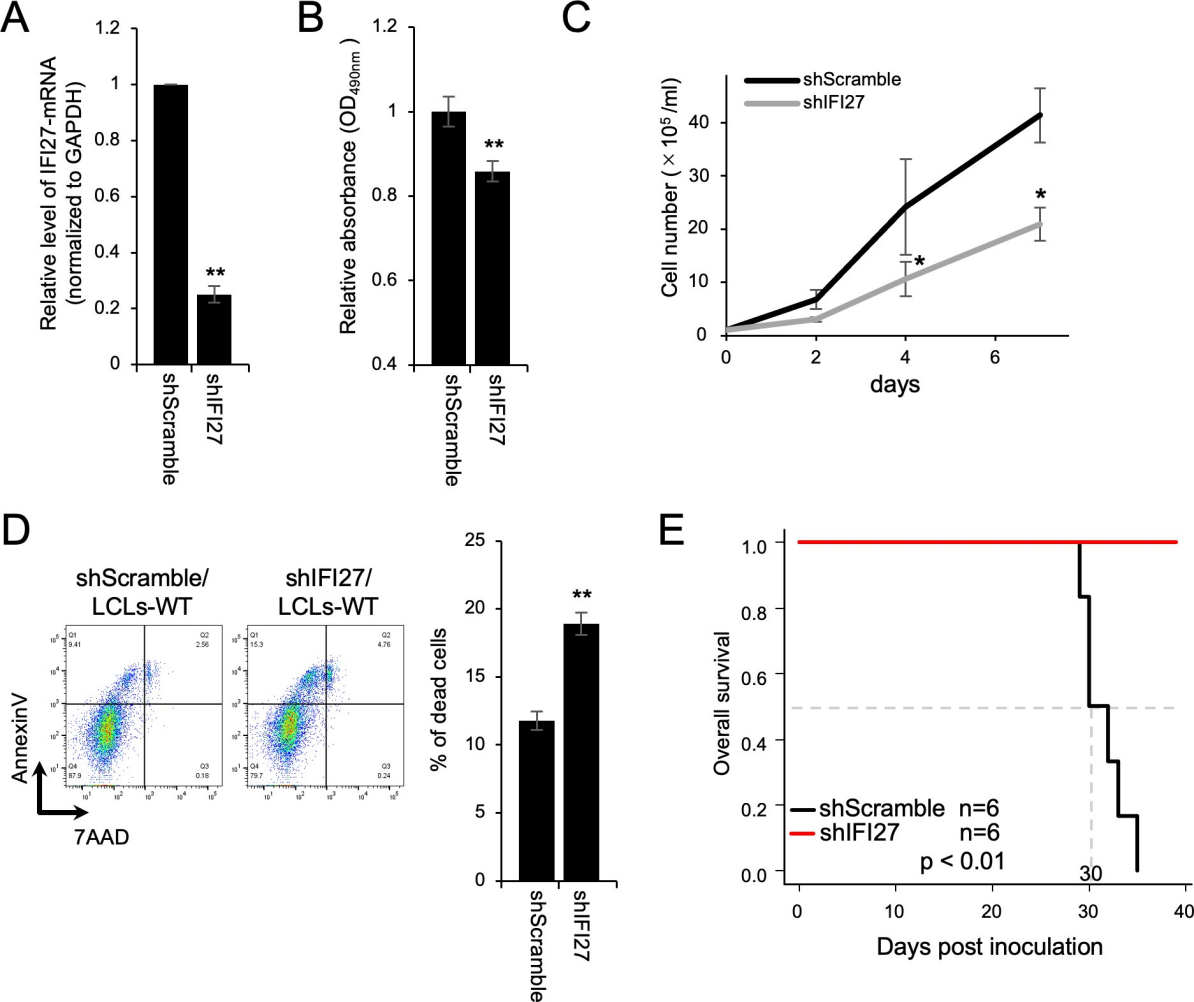
Knockdown of IFI27 impairs LCL survival *in vitro* and *in vivo*. (A) RT-qPCR confirming the knockdown of IFI27 mRNA in LCLs. LCLs-WT carrying shScramble (shScramble/LCLs-WT) and shIFI27 (shIFI27/LCLs-WT) were established by blasticidin selection after lentiviral-mediated shRNA transduction. The results are presented as the mean ± SD. ** p < 0.01. (B) Viability of LCLs-WT carrying shScramble or shIFI27 and cultured for 48 h as assessed by the MTS assay. The results are presented as the mean ± SD. ** p < 0.01. (C) The growth curve of LCLs-WT carrying shScramble or shIFI27 over 7 days after seeding at 2 × 10^5^ cells. The results are presented as the mean ± SD of three independent experiments. * p < 0.05. (D) Annexin V/7-AAD assay of LCLs-WT carrying shScramble or shIFI27. Dead LCLs were defined as those positive for annexin V or both annexin V and 7-AAD. The results are presented as the mean ± SD. ** p < 0.01. (E) Overall survival of 5-week-old mice inoculated with LCLs-WT carrying shScramble or shIFI27. The 50% survival was 30 days in LCLs-WT/shScramble.

We also assessed the effect of IFI27-knockdown (KD) in LCLs-WT. The IFI27 mRNA expression in LCLs-WT expressing shIFI27 (shIFI27/LCLs-WT) was 75% lower than the control level (shScramble/LCLs-WT; Fig 7A). Consistent with effects of IFI27 overexpression (Fig 6), IFI27 knockdown in LCLs-WT significantly decreased the growth rate (Fig 7B and 7C) and increased the rate of cell death (Fig 7D). These findings highlight the role of IFI27 in EBV-transformed cells *in vitro*.

To evaluate the importance of IFI27 to pathogenicity *in vivo*, we inoculated shIFI27/LCLs-WT or shScramble/LCLs-WT into 5-week-old immunodeficient NOG mice intraperitoneally and observed these xenografted mice. As presented in Fig 7E, IFI27-KD increased significantly the survival rate of xenografted mice. These results indicated the roles of IFI27 in tumor development *in vivo*.

### BNRF1-KO or IFI27-KD induced reactive oxygen species production

**Fig 8:**
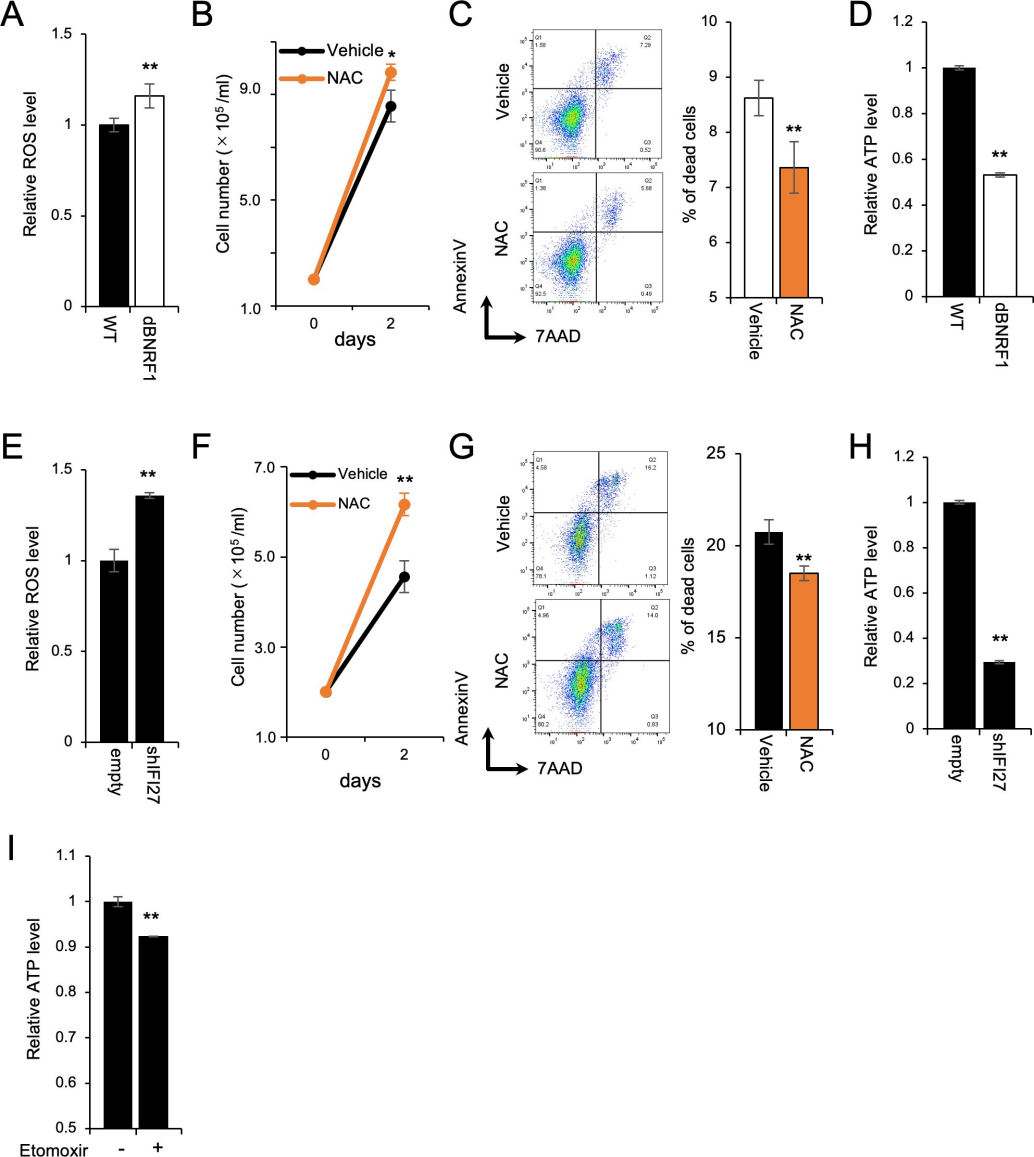
BNRF1-KO or IFI27-KD induced ROS production. (A and E) The relative ROS production of LCLs-WT and LCLs-dBNRF1 (A), or shScramble/LCLs-WT and shIFI27/LCLs-WT (E). The results are presented as the mean ± SD of three independent experiments. ** p < 0.01. (B and F) The growth of LCLs-dBNRF1 (B) or shIFI27/LCLs-WT (F) with or without 2 mM NAC over 2 days after seeding at 2 × 10^5^ cells. The results are presented as the mean ± SD of three independent experiments. * p < 0.05, ** p < 0.01. (C and G) Annexin V/7-AAD assay of LCLs-dBNRF1(C) or shIFI27/LCLs-WT (G) cultured for 48 h in the presence or absence of 2 mM NAC. Dead LCLs were defined as those positive for annexin V or both annexin V and 7-AAD. The results are presented as the mean ± SD. ** p < 0.01. (D and H) The relative ATP production of LCLs-WT and LCLs-dBNRF1 (D), or shScramble/LCLs-WT and shIFI27/LCLs-WT (H). The results are presented as the mean ± SD of three independent experiments. ** p < 0.01. (I) The relative ATP production of LCLs-WT cultured in the presence or absence of 20mM etomoxir. The results are presented as the mean ± SD of three independent experiments. ** p < 0.01.

Recently, IFI27 has been linked to mitochondrial metabolism through fatty acid oxidation (FAO) in adipocytes [29]. During ATP synthesis, mitochondria generate reactive oxygen species (ROS) as an intrinsic by-product [30,31]. Based on the finding that FAO produces higher ROS level than glucose oxidation [32], we assessed ROS production in LCLs-WT and LCLs-dBNRF1. As shown in Fig 8A, the level of ROS was higher in LCLs-dBNRF1. Treatment with N-acetyl cysteine (NAC), an antioxidant, stimulated the growth of LCLs-dBNRF1 and decreased cell death (Fig 8B and 8C), suggesting that ROS were responsible for the fragile growth by BNRF1-KO. Excessive ROS cause insufficient ATP production [33]. Indeed, LCLs-dBNRF1 produced less ATP than LCLs-WT (Fig 8D).

Similar to LCLs-dBNRF1, the KD of IFI27 elicited ROS production (Fig 8E). As expected, NAC rescued the phenotype of shIFI27/LCLs-WT (Fig 8F and 8G). The level of ATP was reduced by IFI27-KD (Fig 8H). These data indicate that the BNRF1-IFI27 axis collateralizes the robust growth of EBV-transformed cells through efficient ATP production and ROS scavenging.

As shown in Fig 8I, ATP production was slightly but significantly reduced by the treatment with etomoxir, an inhibitor of FAO [34], suggesting that FAO was an energy source for LCLs. Simultaneously, we cannot rule out the possibility that IFI27 controls mitochondrial metabolism through not only FAO but also other pathways.

### IFI27 promoted the growth of EBV-infected B cells during primary infection

**Fig 9:**
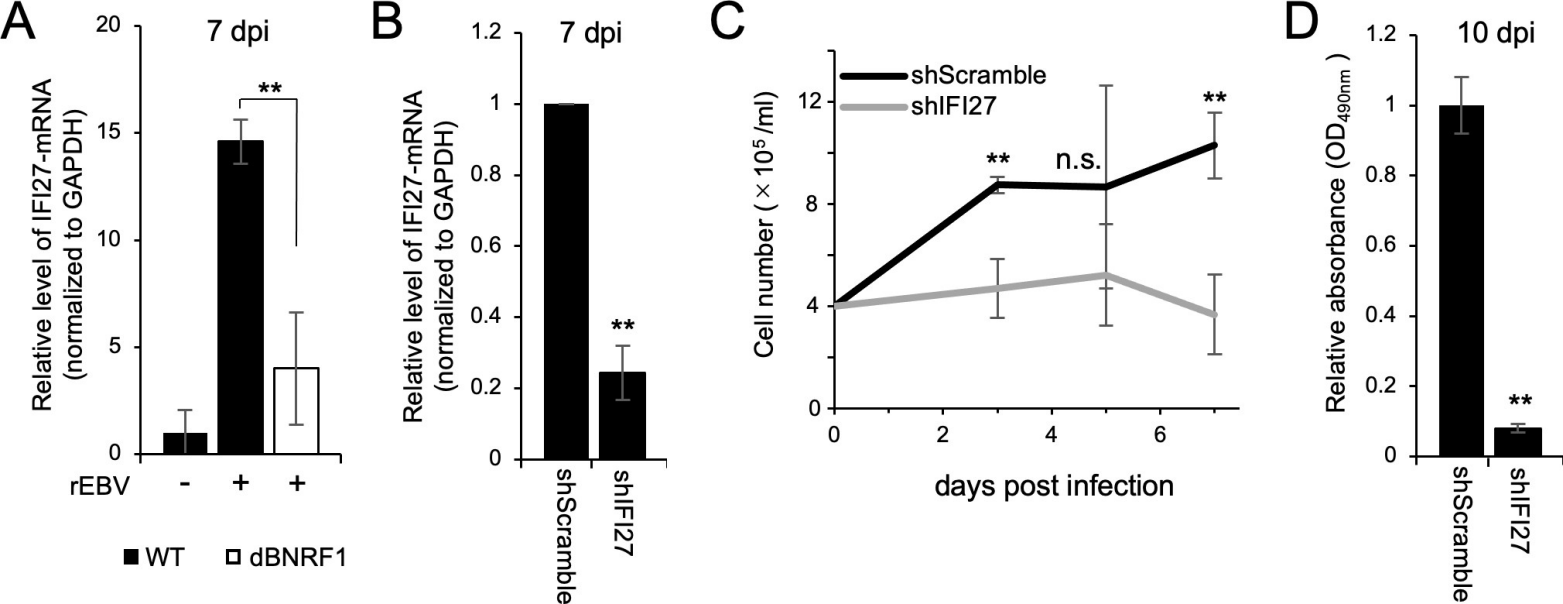
Knockdown of IFI27 impairs the proliferation of EBV-infected B cells during de novo infection. (A) RT-qPCR of IFI27 mRNA in mock-infected B cells or those infected with EBV-WT or EBV-dBNRF1 at 7 dpi. The results are presented as the mean ± SD. ** p < 0.01. (B) RT-qPCR confirming knockdown of IFI27 mRNA in EBV-infected B cells at 7 dpi. The results are presented as the mean ± SD. ** p < 0.01. (C) Growth curve analyses of B cells carrying the indicated shRNA up to 7 dpi with EBV. Cells were seeded at 4 × 10^5^ cells. The results are presented as the mean ± SD. ** p < 0.01. (D) Viability of EBV-infected B cells carrying shScramble or shIFI27 at 10 dpi. The results are presented as the mean ± SD. ** p < 0.01.

The finding that a large amount of BNRF1 contained in virions is transferred to B-cells during EBV infection [7] suggests that the BNRF1-IFI27 axis modulates the growth of EBV-infected cells during primary infection. In fact, recombinant EBV devoid of BNRF1 transformed primary B-cells much less efficiently than EBV-WT [10]. To explore this possibility, we compared the mRNA expression of IFI27 between B-cells infected with EBV-WT and EBV-dBNRF1. As shown in Fig 9A, EBV-dBNRF1 did not induce IFI27 expression 7 days after EBV infection.

Furthermore, we assessed the effect of IFI27 on EBV infection using B-cells expressing shIFI27. IFI27 expression was suppressed in EBV-infected B-cells in the presence of shIFI27 (Fig 9B). The growth curve demonstrated that IFI27-KD inhibited the proliferation of EBV-infected B-cells (Fig 9C). We also confirmed that the growth of EBV-infected B-cells at 10 days post-infection (dpi) was significantly suppressed by shIFI27 (Fig 9D). These findings suggested that BNRF1 protein-mediated IFI27 expression contributed to the growth of infected cells during EBV-mediated transformation.

## Discussion

Accumulating evidence has revealed the role of the EBV lytic cycle in cancer development [6], although the precise mechanisms by which the lytic cycle promotes tumor formation and development remain obscure. Intriguingly, progeny production is not required for these processes [12,20,35,36], indicating that the tumor-associated state of EBV is abortive lytic replication and the lytic genes function not only in genomic replication but also in EBV-driven tumor formation and development. EBV encodes several anti-apoptotic proteins, most of which function in latently infected cells [37]. In this study, we found that abrogation of the EBV major tegument protein BNRF1 resulted in fragile growth in transformed B-cells, leading to a remarkable reduction of the EBV-associated tumor formation in a mouse xenograft model. BNRF1 cell-autonomously induced the expression of IFI27, which ensured robust cell proliferation. In addition, the loss of BNRF1 decreased the transformation activity of primary B-cells as described previously [10]. We also revealed that IFI27 supports the growth of EBV-infected cells during B-cell transformation. The epidemiological findings that pyothorax-associated lymphoma, which is strongly associated with EBV, expresses IFI27 mRNA [38] supports our findings, although the expression of BNRF1 in that lymphoma has not been assessed. Therefore, the BNRF1-IFI27 axis was required for the EBV-mediated tumor formation and development.

IFI27, which is stably induced by type I interferon [39], belongs to the FAM14 family of protein carrying the ISG12 motif [39]. Human IFI27 is considered a transmembrane protein [40,41]. Previous studies demonstrated that IFI27 enhanced DNA-damage induced apoptosis. This pro-apoptotic effect of IFI27 is canceled by Bcl-2 co-expression [39,42]. EBV regulates intrinsic apoptosis in infected cells by inducing Bcl-2 expression via LMP1 [43] and expressing viral Bcl-2 proteins, BHRF1, and BALF1 [44,45]. Over the past decade, IFI27 has been reported to promote tumor cell growth and migration in several cancers [14–19]. Furthermore, recent studies have shown that IFI27 regulates mitochondrial metabolism and thermogenesis in adipocytes [29,46]. Herein, we demonstrated that perturbation of the BNRF1-IFI27 axis impaired ROS scavenging and ATP production (Fig 8). Although IFI27 promotes mitochondrial bioenergetics upon cold stress by facilitating FAO in adipocytes [29], an inhibitor of FAO reduced ATP production in LCLs, but was not completely inhibited (Fig 8I), suggesting that IFI27 controlled mitochondrial metabolism and energy homeostasis in LCLs using not only FAO but also other energy sources. Indeed, the genetic ablation of IFI27 causes broad repression of mitochondrial gene expression [46] and a decrease in the number of mitochondrial cristae [29]. Abnormal mitochondrial morphology is correlated with ROS production [47]. The underlying molecular mechanism by which IFI27 promotes the cell growth and survival of LCLs remains an open question, and further study is required. We speculate that EBV-infected cells require the IFI27-mediated growth resilience to overcome stresses such as anti-viral responses upon primary infection, and hypoxic and hypovascular circumstances in the tumor.

EBV seroprevalence increases with age. Approximately 95% of healthy adults are infected with EBV [48]. BNRF1 is a major EBV antigen in EBV-seropositive healthy donors [49]. Interestingly, CD8+ T cell clones raised against the tegument protein BNRF1 recognize latent growth-transforming B-cells [50], implying the expression of BNRF1 protein in a latent phase. Consistently, we could detect the mRNA encoding BNRF1 in the latently infected B-cells, although the BNRF1 protein was not detected (Figs 3 and S4). It should be noted that BNRF1 is categorized as a late gene in HEK293/EBV cells [51]. Our findings highlighted the role of BNRF1 in the latent cycle in infected B-cells.

We could not eliminate the possibility that BNRF1 proteins were transferred from the occasional lytic-induced cells to the latently infected cells via extracellular vesicles (EVs) such as exosomes because BNRF1 proteins were incorporated into EVs [52].

This study had several limitations. First, we evaluated IFI27 expression by RT-qPCR, but we did not detect endogenous IFI27 protein because of the limitations of commercial antibodies against IFI27. Second, our results must be confirmed using clinical samples. The information on whether IFI27 is upregulated in EBV-associated tumors requires further investigation. Likewise, the downstream process of IFI27-mediated growth should be intensively studied. These findings will shed light on a potential therapeutic target in EBV-driven transformed cells.

In summary, BNRF1, an EBV lytic gene product, supports the survival of latent growth-transforming B-cells infected with EBV via upregulation of IFI27 both *in vitro* and *in vivo*. BNRF1-KO or IFI27-KD decreased the pathogenicity of LCLs in a mouse xenograft model. Our findings provided insights into the growth resilience of EBV-infected cells via the BNRF1-IFI27 axis.

## Methods

### Ethics statement

The study was approved by the Institutional Review Board of Nagoya University Hospital (number 2022-32-2). Written informed consent was obtained from all participants individually before the survey. All animal experiments were approved by the University Committee (number M220193-004) under the Guidelines for Animal Experimentation at Nagoya University.

### Cell culture

HEK293T and HEK293T/EBV cells were grown in DMEM (Sigma-Aldrich, St. Louis, MO, USA) supplemented with 10% FBS. Akata(-) cells, Akata/EBV-EGFP [53], and LCLs established by recombinant EBV infection were maintained in RPMI 1640 supplemented with 10%-15% FBS. AGS/EBV-EGFP cells (kindly gifted by Hironori Yoshiyama) [54] were grown in RPMI 1640 medium containing 10% FBS and 750 μg/mL G418 [55].

### Plasmids

The expression vectors pcDNA-BZLF1 and pcDNA-gB were reported previously [56]. Lentiviral expression constructs of tetracycline-inducible BNRF1-HA, shRNA for IFI27, and control shRNA (pLV-Tet3G and pLV-TRE-BNRF1-HA, pLV-shIFI27-T2A-mCherry, and pLV-shScramble-mCherry) were generated by VectorBuilder (Chicago, IL, USA). To express C-terminal HA-tagged BNRF1 (BNRF1-HA) or Flag-tagged IFI27 (IFI27-Flag), the fragments were cloned into the CSII-CMV-MCS-IRES2-Bsd vector (a gift from Dr. Hiroyuki Miyoshi, RIKEN BioResource Center, Wako, Japan). The inserted DNA sequence of each vector was confirmed by direct DNA sequencing.

### Construction of the dBNRF1 EBV-BAC genome

The original EBV-BAC (B95-8 strain) was kindly provided by Dr. W. Hammerschmidt [57]. To construct dBNRF1-rEBV and revertant dBNRF1rev-rEBV, homologous recombination was performed in *Escherichia coli* to generate the C429A mutation in the BNRF1 ORF and restore the wild-type sequence, as described previously [56]. The oligonucleotides used for the series of recombination are presented in Table 1. The targeted recombination and full bacmid sequence were confirmed by Sanger and Nanopore sequencing, respectively (Eurofins Genomics Japan, Tokyo, Japan). HEK293T cells were transfected with recombinant EBV using Fugene 6 reagent (Promega, Wisconsin, USA) and cultured with 150 μg/mL hygromycin B (Takara, Shiga, Japan). After 2 weeks post-transfection, hygromycin-resistant and green fluorescent protein (GFP)-positive cell colonies were cloned as HEK293T/EBV lines for further analyses.

**Table 1:** Oligonucleotides used for the generation of recombinant EBV.

Oligo name	Sequence (5’ to 3’)
BNRF1-NeoSt-pF	GGCCCTCGTTGGCATTCTACTAGGAAACGGCGACAGGGTGAACACTTGGGCACGGAGAGGGCCTGGTGATGATGGCGGGATC
BNRF1-NeoSt-pR	CAAGTGGCCCGAGTAAGTGTCTCGCAGCGCGGACACGATCTTAGCTCGTCGGCCAGCTGTCGGAAGAACTCGTCAAGAAGG
BNRF1-stop-pF	GGCGCCACGTAAGTGCTTCGCG
BNRF1-stop-pR	CGCGAAGCACTTACGTGGCGCC
BNRF1-rev-pF	GGCCCTCGTTGGCATTCTAC
BNRF1-rev-pR	CAAGTGGCCCGAGTAAGTGTC

### Establishment of LCLs

HEK293T cells having recombinant EBV were transfected with the BZLF1 and gB expression plasmids using polyethylenimine (Polysciences, Warrington, PA, USA). Three days after transfection, supernatants from HEK293T/WT-rEBV, HEK293T/dBNRF1-rEBV, or HEK293T/dBNRF1rev-rEBV were harvested; passed through 0.45 μm filters; ultracentrifuged at 100,000 × g for 1.5 h; and used as a virus stock. EBV-negative Akata(-) cells were infected with the virus, and GFP-positive cells were counted using Fortessa X-20 (Becton Dickinson, Franklin Lakes, NJ, USA) to measure the viral titer. LCLs-WT, LCLs-dBNRF1, and LCLs-dBNRF1rev were established as described previously [20].

### Lentiviral transduction

Lentiviruses for LCLs were produced by co-transfecting HEK293T cells with pCMVR8.74 (a gift from Dider Trono and Yasuo Ariumi; #22036, Addgene, Watertown, MA, USA), phCMV-GALV-MTR (a gift from Daniel Hodson; #163612, Addgene), and a third plasmid (CSII-BNRF1-HA, CSII-CMV-MCS-IRES2-Bsd, CSII-IFI27-Flag, pLV-shIFI27-T2A-mCherry, or pLV-shScramble-mCherry). Lentiviruses for Akata(-) cells were produced by co-transfecting HEK293T cells with pCMVR8.74, pCMV-VSV-G (a gift from Bob Weinberg; #8454, Addgene), and pLV-Tet3G or pLV-TRE-BNRF1-HA.

LCLs were infected with the lentiviruses by spinoculation at 1500 × g for 1.5 h in the presence of 5 μg/mL polybrene (VectorBuilder). After incubation for 3 h, LCLs were resuspended in a fresh medium. At 3 dpi, infected LCLs were incubated with 10 μg/mL blasticidin for at least 10 days.

To establish Tet-BNRF1-HA/ Akata(-) cells inducibly expressing BNRF1-HA, Akata(-) cells were infected with a lentivirus carrying the Tet3G cassette in the presence of 5μg/mL polybrene, and the next day, the culture medium was replaced with fresh medium containing 150 μg/mL hygromycin. After 14 days of culture, cells were infected with a lentivirus carrying the TRE-BNRF1-HA cassette as previously described, and maintained in the presence of 10 μg/mL blasticidin and 150 μg/mL hygromycin.

### EBV infection in shRNA-transduced B cells

Primary B cells were isolated using EasySep human CD19 positive selection kit II (Veritas, Tokyo, Japan) from healthy donor PBMCs according to the manufacturer’s instructions. Isolated B cells were infected with lentiviruses by spinoculation at 1,500 × g for 1.5 h on plates coated with RetroNectin according to the manufacturer’s instructions (Takara). Three hours after spinoculation, cells were infected with EBV-EGFP [58] at a multiplicity of infection of 1. Infected B cells were incubated with 10 μg/mL blasticidin at 3 dpi.

### Antibodies, immunoblotting, and flow cytometry

Anti-BNRF1 was kindly provided by Dr. Lieberman [59], and anti-BZLF1 antibody was purchased from Santa Cruz Biotechnology (Santa Cruz, CA, USA). Polyclonal antibodies against BMRF1, BALF4 (glycoprotein B), and LMP1 were described previously [56]. Anti-STAT1 (#9172), anti-phospho-STAT1 (Tyr701) (#9171), and anti-GAPDH (#5174) antibodies were obtained from Cell Signaling Technology (Danvers, MA, USA). Anti-STAT2 (#693302) and anti-phospho-STAT2 (Tyr631) (#619851) antibodies were purchased from Biolegend. Anti-HA antibody (3F10) (#11867423001)and anti-Flag antibody (M2) (F1804) were purchased from Sigma-Aldrich.

Immunoblotting was performed as described previously [60]. Densitometry was performed using ImageJ.

For surface staining, cells were incubated with anti-glycoprotein B antibody before fixation. Then cells were stained with Alexa 647-anti-mouse IgG (A-21235; ThermoFisher Scientific, Waltham, USA) on ice for 30 min. Antibody-stained cells were fixed overnight with 4% paraformaldehyde at 4°C. Subsequently, cells were treated with 0.1% Triton-X100/PBS at room temperature for 10 min. Cells were then stained further with PE-anti-BZLF1 antibody (sc-53904 PE; Santa Cruz Biotechnology) on ice for 30 min. Cells were analyzed using a BD Fortessa X-20.

### Annexin V/7-AAD assay

The death of LCLs was evaluated by flow cytometry using allophycocyanin annexin V (Biolegend, San Diego, CA, USA) and 7-AAD (Becton Dickinson) according to the manufacturer’s instructions.

### Cell viability assay (MTS assay)

Cell viability was measured using Cell Titer 96 Aqueous One Solution (MTS reagent; Promega) as described previously [61]. The absorbance was measured at 490 nm on a Rainbow plate reader (Tecan Japan, Kawasaki, Japan).

### Quantification of viral DNA

Viral DNA in replicating cells or whole blood of NOG mice was quantified by quantitative real-time PCR (qPCR) as described previously [62].

### RT-qPCR

Total RNA was purified using TriPure isolation reagent (Sigma-Aldrich) according to the manufacturer’s instructions. Total RNA was subjected to RT-qPCR using One Step TB Green PrimeScript RT-PCR Kit II (Takara) and real-time PCR system 7500 Fast Dx (ThermoFisher Scientific). The primers used for RT-qPCR are presented in Table 2.

**Table 2:** Primers used for RT-qPCR.

Primer name	Sequence (5’ to 3’)
IFI27-F	CGTCCTCCATAGCAGCCAAGAT
IFI27-R	ACCCAATGGAGCCCAGGATGAA
BNRF1-F	CAGAGACCGCTGACACGAGG
BNRF1-R	CTGAAGGACCAAGTGGCCCG
GAPDH-F	GTCTCCTCTGACTTCAACAGCG
GAPDH-R	ACCACCCTGTTGCTGTAGCCAA

### RNA sequence

Tet-BNRF1/Akata(-) cells cultured with or without 1μg/mL Dox for 2 days, LCLs-WT, and LCLs-dBNRF1 were harvested and total RNA was extracted using an RNeasy mini kit (Qiagen, Hilden, Germany). The evaluation of RNA quality, RNA-seq library preparation, Illumina sequencing, and data preprocessing were performed as described previously [20].

### B cell transformation assay

The transformation assay was performed as described previously [63].

### Xenograft experiments using LCLs

Either five- or six-week-old female NOG mice (Central Institute for Experimental Animals, Kawasaki, Japan) were inoculated intraperitoneally with 2 × 10^5^ LCLs suspended in 200 μL of phosphate-buffered saline. Mice survival was the primary endpoint, and mice were sacrificed according to ethical guidelines if their weight decreased by more than 15% versus the basal weight, remarkable ruffled fur was observed, all mice of either group died, or mice were alive on day 70 after LCL inoculation, whichever came first. Tumor formation was assessed in all mice at autopsy.

Immunohistochemical staining of LMP was performed using an anti-LMP antibody (M0897; Agilent, Santa Clara, CA, USA) and a Leica BOND-MAX (Leica, Bannockburn, IL, USA) with BOND Polymer Detection (ds9800; Leica). During the blocking phase, endogenous mouse tissue IgG was blocked by incubation with an anti-IgG antibody (ab6668; Abcam, Cambridge, UK) at a concentration of 0.1 mg/mL at room temperature for 1 h. EBER-ISH was performed in Kotobiken Medical Laboratories (Tokyo, Japan) as described previously [20].

### Intracellular ROS and extracellular ATP assays

Cellular ROS levels were measured using the Cellular ROS Assay kit (ab186029; Abcam) in accordance with the manufacturer’s instructions. Extracellular ATP levels were measured using an ATP Assay Kit-Luminescence (#346–09793; Dojindo, Kumamoto, Japan) in accordance with the manufacturer’s instructions, with a SpectraMax id5 (San Jose, CA). *N*-Acetyl-L-cysteine (A9165; Sigma-Aldrich) was purchased from Merck (Darmstadt, Germany). Etmoxir (#11969) was purchased from Cayman Chemical (Ann Arbor, MI, USA)

### Statistical analysis

Continuous variables were tested using Student’s t-test. Survival analyses were conducted by the log-rank test using EZR version 1.36 (Saitama Medical Center, Jichi Medical University, Saitama, Japan) [64]. A two-sided P value of < 0.05 indicated statistical significance.

## Supporting information

S1 Fig Summary of full bacmid sequences. (A) Whole sequence of rEBV-WT (upper), rEBV-dBNRF1 (middle), and rEBV-dBNRF1rev (lower). The lowest column shows the coding genes of EBV. Colored lines indicate a point mutation in each read compared with the reference sequence. (B) Sequence surrounding the BNRF1 locus.
(TIFF)

S2 Fig EBV-DNA in peripheral blood of mice inoculated with LCLs. The EBV copy number in peripheral blood of mice inoculated with LCLs-WT and LCLs-dBNRF1 was quantified by qPCR analysis at the indicated time points.
(TIFF)

S3 Fig Pathogenicity of LCLs in 5-week-old NOG mice. (A) Overall survival for mice inoculated with LCLs-WT or LCLs-dBNRF1. The time for 50% survival was 28 days for LCLs-WT and 35 days for LCLs-dBNRF1. (B) Histochemistry of the intraperitoneal tumors stained with hematoxylin and eosin (top), and analyzed by EBER in situ hybridization (middle) and LMP1 immunohistochemistry (bottom). The images shown are representative of two independent experiments with similar results. Scale bar, 100 μm.
(TIFF)

S4 Fig Immunoblots of LCLs using an anti-BNRF1 antibody. Lysates from HEK293T/EBV-WT transfected with pcDNA-BZLF1 and indicated LCLs were analyzed by immunoblotting with the BNRF1 antibody.
(TIFF)

S5 Fig Effects of BNRF1-KO on gene expression of LCLs. (A) RNA-seq volcano plot analysis of differentially expressed genes (DEGs) in LCLs-WT compared with LCLs-dBNRF1. Upregulated and downregulated DEGs are mapped as red and blue spots, respectively. (B and C) Gene ontology biological process enrichment analysis of DEGs that were upregulated (B) and downregulated (C) in LCLs-WT compared with LCLs-dBNRF1.
(TIFF)

S6 Fig Temporal changes in IFI27 and BNRF1 gene expression during primary EBV infection in B cells. The heatmap is generated from DRA011328 in the DNA Data Bank of Japan. The heatmap shows normalized Z score for each gene and the colors indicate an increase (or decrease) in gene expression.
(TIFF)

S1 Table Percentage of BZLF1- or BZLF1- and gB-double positive cells in each indicated LCLs. (XLSX)

S2 Table Significant differentially expressed genes in LCLs-WT compared with LCLs-dBNRF1 in RNA-seq analyses. (XLSX)

S3 Table EBV-encoded genes with a log_2_ fold-change in LCLs-WT compared with LCLs-dBNRF1 in RNA-seq analyses. (XLSX)

S4 Table Log_2_ fold-change of genes with rpkm > 1 in a Tet-BNRF1/Akata(-) cells in the presence or absence of Dox in RNA-seq analyses. (XLSX)
