# Supplementary figures and images for: Epstein-Barr virus lytic gene BNRF1 promotes B-cell lymphomagenesis via IFI27 upregulation
Source: PLoS Pathog. 2024 Feb 1;20(2):e1011954. doi: 10.1371/journal.ppat.1011954 (PMC10833513; doi:10.1371/journal.ppat.1011954)

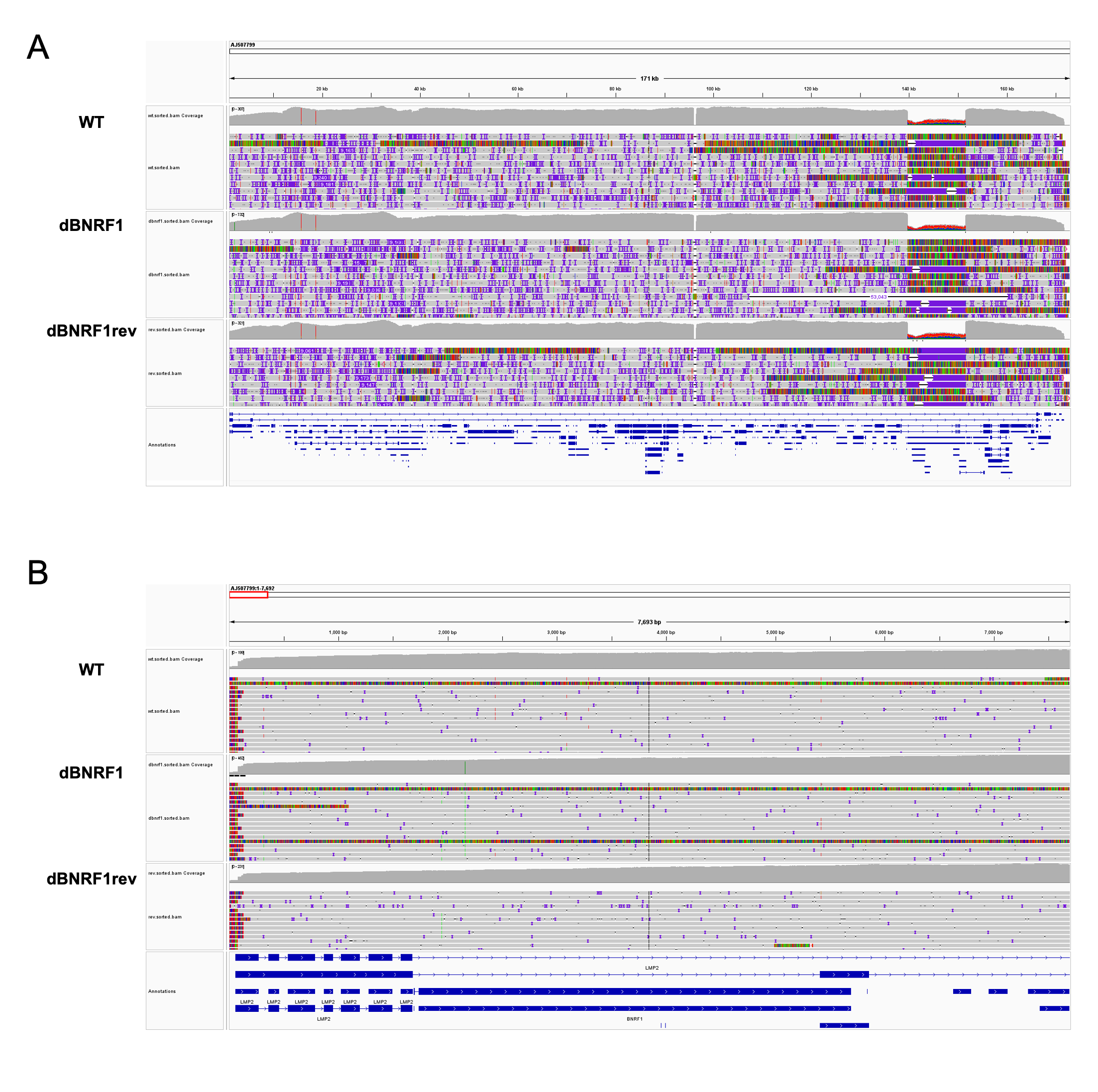

Supplement: S1 Fig — (A) Whole sequence of rEBV-WT (upper), rEBV-dBNRF1 (middle), and rEBV-dBNRF1rev (lower). The lowest column shows the coding genes of EBV. Colored lines indicate a point mutation in each read compared with the reference sequence. (B) Sequence surrounding the BNRF1 locus. (TIFF) [file ppat.1011954.s001.tiff]

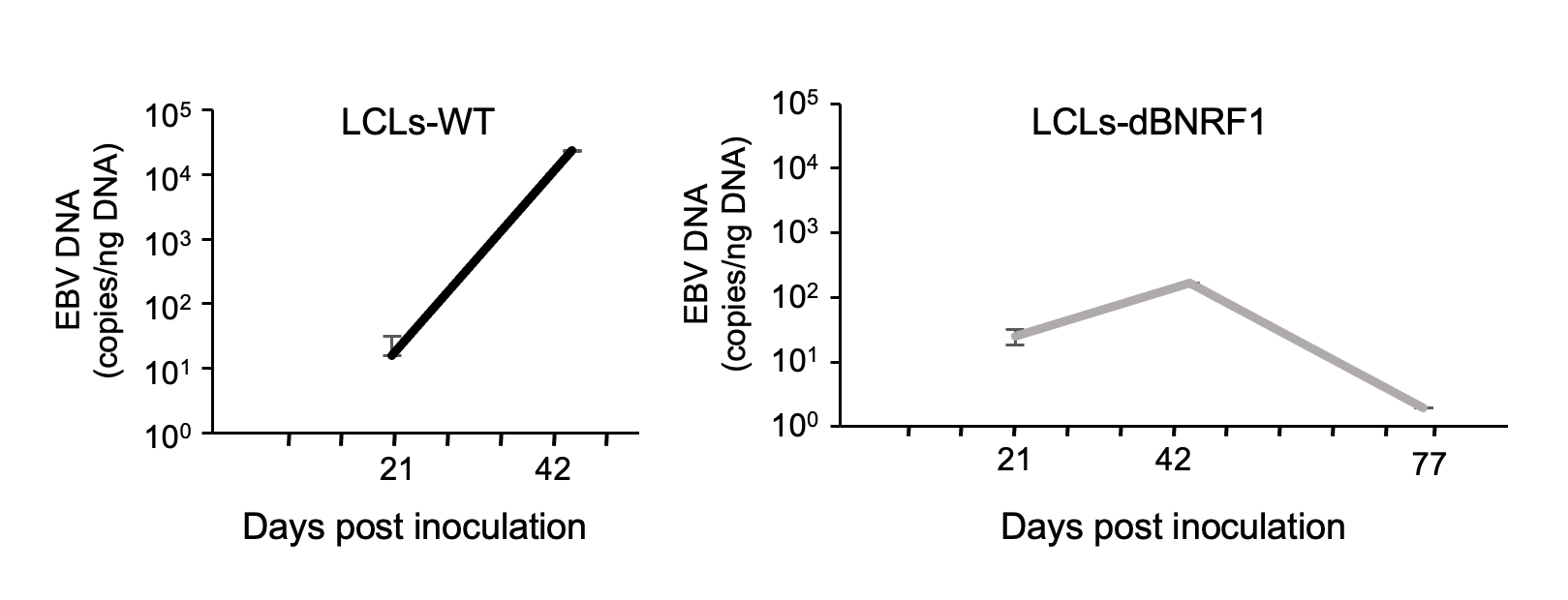

Supplement: S2 Fig — The EBV copy number in peripheral blood of mice inoculated with LCLs-WT and LCLs-dBNRF1 was quantified by qPCR analysis at the indicated time points. (TIFF) [file ppat.1011954.s002.tiff]

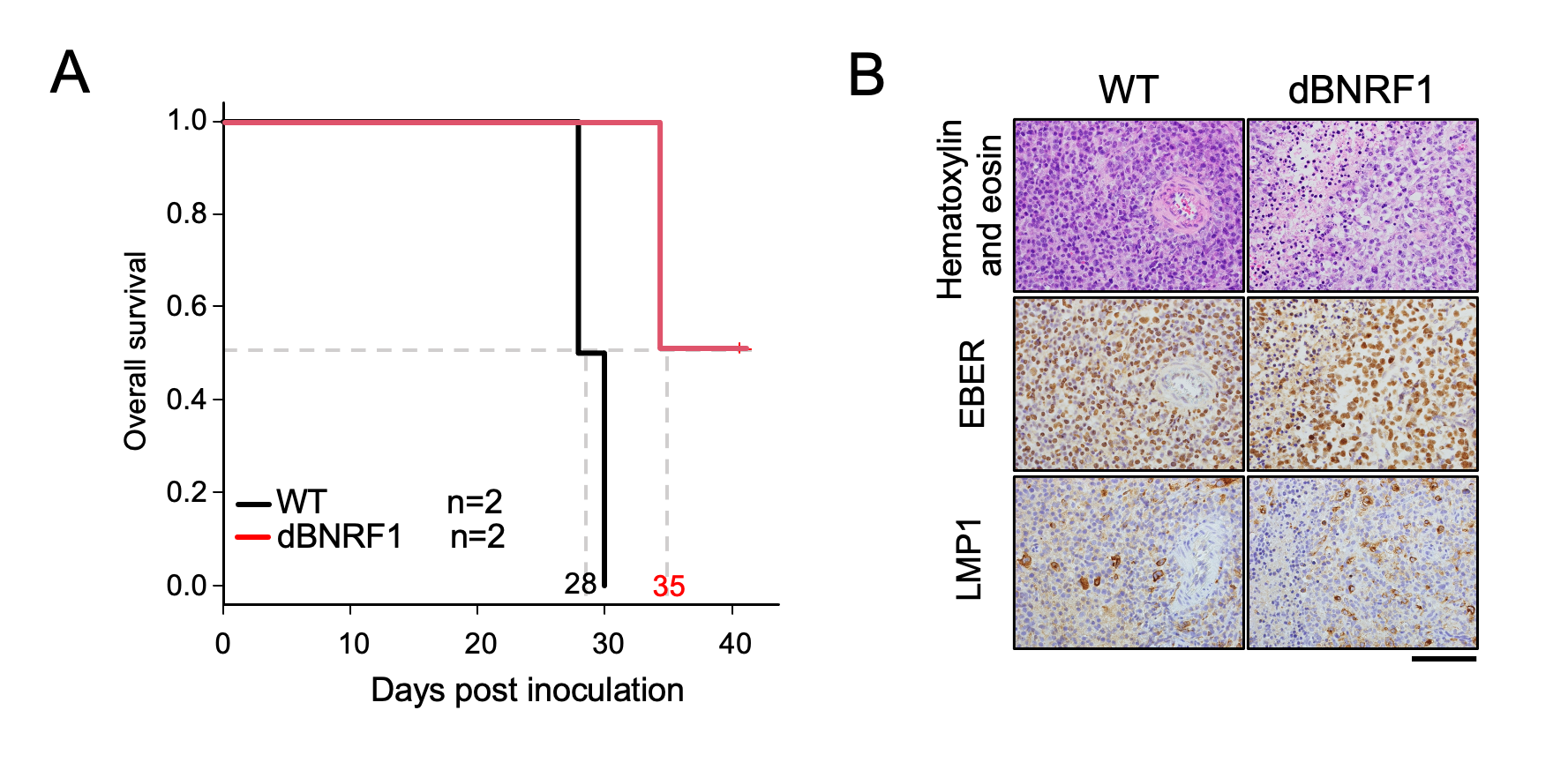

Supplement: S3 Fig — (A) Overall survival for mice inoculated with LCLs-WT or LCLs-dBNRF1. The time for 50% survival was 28 days for LCLs-WT and 35 days for LCLs-dBNRF1. (B) Histochemistry of the intraperitoneal tumors stained with hematoxylin and eosin (top), and analyzed by EBER in situ hybridization (middle) and LMP1 immunohistochemistry (bottom). The images shown are representative of two independent experiments with similar results. Scale bar, 100 μm. (TIFF) [file ppat.1011954.s003.tiff]

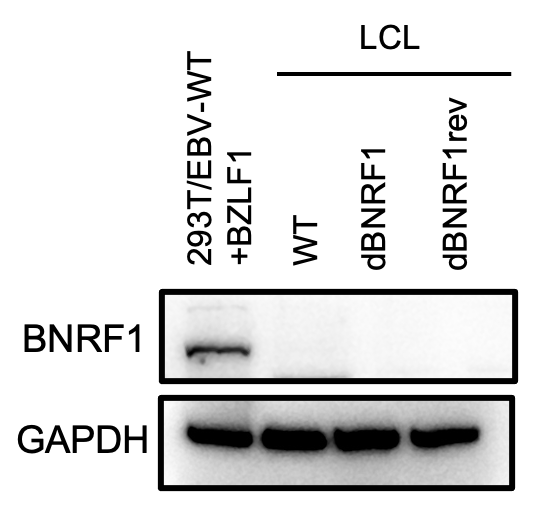

Supplement: S4 Fig — Lysates from HEK293T/EBV-WT transfected with pcDNA-BZLF1 and indicated LCLs were analyzed by immunoblotting with the BNRF1 antibody. (TIFF) [file ppat.1011954.s004.tiff]

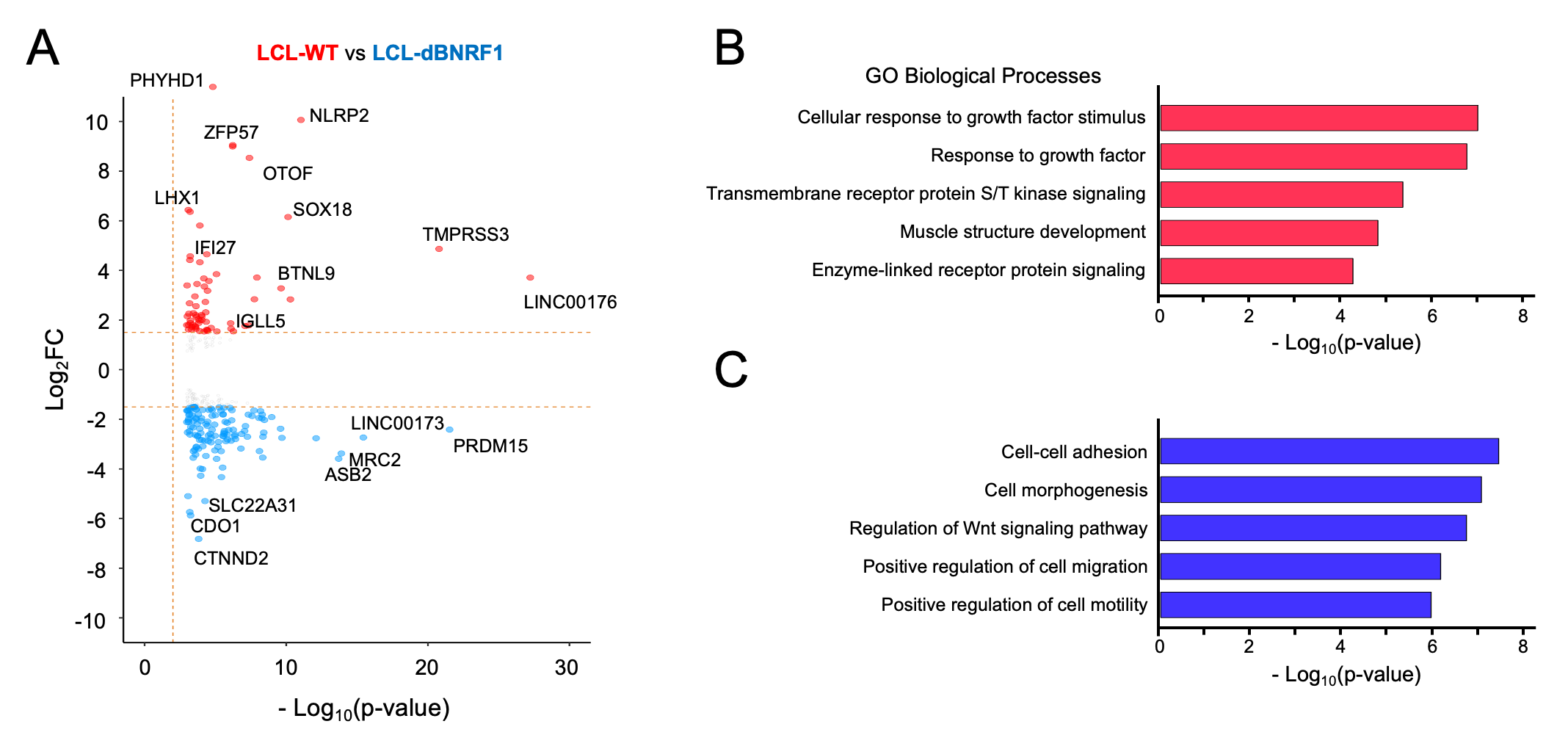

Supplement: S5 Fig — (A) RNA-seq volcano plot analysis of differentially expressed genes (DEGs) in LCLs-WT compared with LCLs-dBNRF1. Upregulated and downregulated DEGs are mapped as red and blue spots, respectively. (B and C) Gene ontology biological process enrichment analysis of DEGs that were upregulated (B) and downregulated (C) in LCLs-WT compared with LCLs-dBNRF1. (TIFF) [file ppat.1011954.s005.tiff]

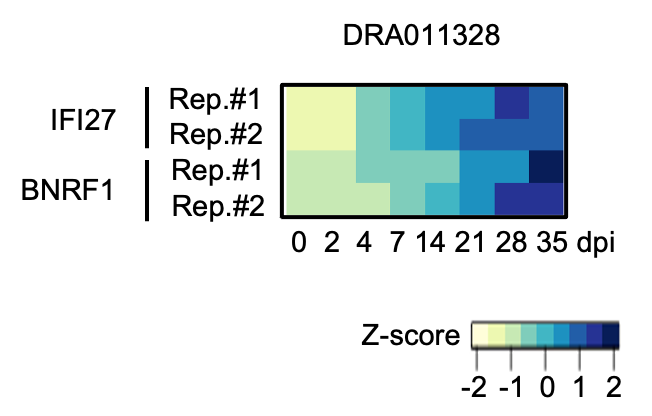

Supplement: S6 Fig — The heatmap is generated from DRA011328 in the DNA Data Bank of Japan. The heatmap shows normalized Z score for each gene and the colors indicate an increase (or decrease) in gene expression. (TIFF) [file ppat.1011954.s006.tiff]
